# Supplementary material for: Time separating spatial memories does not influence their integration in humans
Source: PLoS One. 2023 Aug 10;18(8):e0289649. doi: 10.1371/journal.pone.0289649 (PMC10414573; doi:10.1371/journal.pone.0289649)
Supplement: S1 File — (PDF) [file pone.0289649.s001.pdf]

Supporting Methods

Preregistered sequential analyses and stopping procedures

The target sample size and stopping rules were determined in Wave 1, comparing the 3h to 27h delay conditions. A power analysis indicated that n = 100 per group was needed to achieve 80% power to detect an effect of Cohen's d = 0.40—the smallest effect size across both previous reports on the influence of temporal delay on memory integration [1,2]; Cohen's d = 0.40 – 0.50). Given the high cost and workload required to run the full sample, we took a sequential analysis approach to allow for the possibility that data collection could be terminated early [3] at four predetermined interim checkpoints (at n = 44, 60, 80, 100 per group). We planned to terminate data collection at a given checkpoint if either (a) there was a significant effect of temporal delay at an alpha level adjusted to avoid Type 1 error inflation or (b) there was strong Bayesian evidence for the null ($BF_{01} > 5$). At the first interim analysis in Wave 1, we found strong evidence for the null and therefore terminated data collection. The sample size of the 30min condition in Wave 2 was predetermined to match the conditions from Wave 1. In the final analysis, our sample size became 42 for the 27h condition after excluding data from participants who had computers with low temporal resolution (n=2; see *Data acquisition and preprocessing* below); however, we did not collect more data as we observed strong evidence for the null effect of temporal delay on memory differentiation and memory integration.

Practice task procedures

The practice tasks were designed to mimic all the components of the full learning task but was performed in a perceptually distinct virtual environment (octagon arena situated within a town; see **Fig S1B**). As in the main task, participants searched for coins in points drawn from a Gaussian distribution centered in a quadrant. The practice task also included three phases, mirroring the learning tasks: Environment Familiarization, Search Familiarization, and Reward Searching. Each is described below.

Environment Familiarization. This phase used the same procedures as in the main experimental task.

Search Familiarization. This phase used the same procedures as in the main experimental task.

Reward Searching. This phase was an abbreviated version of the reward searching phase from the main experimental task with a few differences. First of all, four rather than six points were drawn from the Gaussian distribution. Secondly, instead of 37 trials, 12 feedback

trials were interspersed with four no-feedback trials in the same way as in the learning sessions of the main task. Finally, different from the main experiment, the font colour changed when participants collected more than 20 coins in the no-feedback trials to scaffold performance. To ensure all participants were capable of navigating virtual environments, they were required to collect at least 20 coins per trial in four of 16 possible trials to progress to the learning tasks. The practice session terminated when participants reached this criterion or when they completed all 16 trials.

Data acquisition and preprocessing

Participants completed the tasks by running lab-developed applications on their own computers, as described in [4]. This required us to be more cautious compared to running in-lab studies. Prior to the first session, we (1) asked participants to make sure their computers had available storage of at least 300MB to download and run the applications; and (2) checked in with participants about their computer specifications to make sure our applications were likely to work on their devices. Based on our experiences, applications typically ran smoothly on computers with at least 4GB of memory. While most devices that met these requirements worked well, we did encounter situations where a computer met the memory requirement, but the processor was too old to run the applications, and therefore, a session had to be terminated. During the sessions, participants shared their screens via Zoom so that experimenters could monitor the whole process and provide help when needed. They (1) instructed participants to close applications that were irrelevant to the experiment to maximize memory allocation for the experimental applications and to minimize distractions at the beginning of a session; (2) guided participants to download and install the specific version of our experiment application that was compatible with their operating systems (Windows or Mac OS); (3) checked in with participants during the practice task and breaks to make sure they (a) did not experience lags, which were typically related to poor computer performance, and (b) were remaining engaged in the tasks.

The experiment recorded participants' coordinates in the arenas at each time point, as well as their location whenever a coin was collected. The temporal resolution of raw data ranged from 6.17-28.42 Hz, depending on the specifications of the participant's own computer. To ensure that data was directly comparable across participants with different sampling rates, we resampled all data to 10 Hz. We excluded participants whose computers had outlying sampling rates below this rate (n=2), as this would signify low data quality. For trials with fixed one-minute durations, we discarded time points beyond time limits, as a trial sometimes ran over time, likely due to computer specifications (0.76% of trials lasted longer than 62 seconds). We further

discarded the first five seconds of each trial when participants were mostly reorienting themselves before they started moving in an arena. Finally, we took out time points when participants were not moving. After these preprocessing steps, we computed the proportion of all recorded locations spent within each of the four zones (Target, Alternate, Adjacent, and Control) as our primary dependent measure (i.e., time-in-zone).

Measuring learning

Learning performance was operationalized as the average proportion of time spent in the target zone across the last two no-feedback trials in each arena's learning phase. We only considered the final two no-feedback trials because they occurred after participants experienced all six reward points within the larger distribution, and therefore performance in those trials should reflect experience with the complete reward distributions. Further, we excluded feedback trials because participants could use concurrently presented feedback to identify reward locations without any need for memory, thereby inflating the estimate of learning. In the later analyses, participants' learning in the first session was included as a covariate to control for individual differences relevant to spatial learning.

We additionally considered learning speed in some exploratory follow-up analyses. We operationalized learning speed as the average proportion of time spent in the target zone across all no-feedback trials within each learning phase. Our logic was that participants who quickly learned the reward locations and continued to search in them throughout the learning phase would have a higher score across the entire phase, whereas those who were slower to achieve the same ultimate level of performance would have a lower score when averaged across the phase.

Measuring memory integration and differentiation during the Direct test (preregistered confirmatory + exploratory analyses)

As preregistered, memory differentiation was quantified as the difference in time spent in the Target vs. Alternate Zones (Target – Alt), with a positive value indicating that participants differentiated between the two, and a value close to 0 indicating that they did not differentiate between them. As an exploratory complement to the differentiation score, we quantified memory integration across arenas as the difference in time spent in the Alternate and Adjacent Zones (Alt – Adj), which are equated for distance from the Target Zone. A more positive value indicates that participants integrated their memories across sessions because they were biased to search in the other arena's reward locations. Finally, we compared the difference between

time spent in the Adjacent and Control Zones (Adj – Ctrl) in an exploratory analysis, for completeness (results presented in **Table S2** and visualized in **Fig 2B**) The three participants who showed the most "integrated" and least differentiated memories in fact misremembered (i.e., swapped) the reward locations of the two arenas instead of actually integrating the memories, as they searched mostly in Alternate Zone regardless of arena. We excluded these participants in the analysis of Direct and Transfer tests, but report results including them in **Fig S5**.

Measuring memory generalization during the Transfer test (preregistered exploratory analyses)

The two trained (circle, square) and three novel morph arenas were included in the Transfer test. For each arena, we calculated the difference in time spent in the area corresponding to the Target Zone of the circle arena versus the square arena. A more positive value reflects greater circle Target Zone searching, whereas a negative value reflects greater square Target Zone searching. If memories are differentiated into two attractor states, considering search behaviour as a function of arena shape should reveal that participants first search in one rewarded zone and then abruptly shift to the other. This behaviour would be best captured by a sigmoid model (or step function). In contrast, participants with integrated memories should more gradually shift their search behaviour across arenas, progressively moving toward the most relevant Target Zone. This pattern would be best fit as a roughly linear function of arena shape. In the extreme, participants would search in the same location regardless of arena shape, a pattern also well fit by a linear model.

Of note, an insensitivity to arena shape, however, could also reflect poor memory for both arenas. Therefore, we ensured each participant retained memories from at least one learning session before analyzing their Transfer test data. To do this in the least biased way possible, we defined successful retention as searching in the Control Zone (i.e., the zone that was never rewarded and was not close to the Target zone) during the Direct test *less* often than during random walking, where random walking was defined for each participant based on their exploration phase averaging across five trials in Arena 1 (i.e., before any coins were collected). Participants whose search time in the Control Zone during the Direct test was less than 5% of the random walking distribution of the corresponding arena (Circle = 0.10, Square = 0.07) were included in the analysis (total n = 110: 30min = 34, 3h: n = 41, 27h: n = 35; χ^2 (2) = 0.78, p = .676), as we inferred from this behavioural profile that they had some memory for the arena. To ensure the results were not driven by participants who remembered only one of the two

arenas, we additionally performed this analysis restricting to participants who exhibited memory for both arenas (total n = 78: 30min = 20, 3h: n = 32, 27h: n = 26; χ^2 (2) = 2.77, *p* = .250), using the same analysis pipeline including the handling of influential points using Cook's distance.

We followed approaches previously used with a similar paradigm [5] to fit individual participants' data to both linear and sigmoid models. Prior to model fitting, we ensured a data range of 0 and 1 through linear transformation using Equation (1):

$$(1).\ Difference = \frac{(TIZ_{Target|Circle} - TIZ_{Taeget|Square}) + 1}{2}$$

We fit data from individual participants with a sigmoid or a linear model using maximum likelihood estimation. In the linear models (Equation 2), *a* represents slope and *b* represents the intercept. In the sigmoid models (Equation 3), *a* determines amplitude, *b* represents horizontal offset, and *c* represents steepness or slope of the curve. We fit individual participants with sigmoid models and linear models separately. Due to differences in the number of parameters (i.e., model complexity) between the two types of models, we used Bayesian information criterion (BIC) values as our index of model fit.

(2) Linear model: $y_i = ax_i + b$

(3) Sigmoid model: $y_i = \frac{a}{1+e^{-c*(x_i - b)}}$

We then calculated the difference in BIC values for linear and sigmoid models for each participant, with a more positive value reflecting that the sigmoid model provided a better fit than the linear model. See Supplemental **Fig S6** for a visualization of most linear vs. most sigmoid curves.

To validate our two measures of differentiation, we related the relative model fit from the Transfer test with our memory differentiation index from the Direct test using linear regression. After that, we examined the effect of temporal delay on the relative model fit. Influential points were identified in each analysis using Cook's distance (threshold: 4/n, n = sample size) and excluded from the main analysis presented in **Fig 3** (see **Fig S7** for results including influential points and participants who swapped the reward locations).

Measuring Session 1 memory reactivation during Session 2 learning (exploratory analyses)

We examined the reactivation and use of first session learning to guide initial searching in the second session. Specifically, we assessed participants' search behaviour in the Search Familiarization phase in Session 2 learning, restricting data to timepoints occurring before participants collected their first coin (i.e., before experiencing the new reward distribution). Reactivation of the Session 1 memory was quantified as the difference between time spent in the now-Alternate Zone (which had been previously rewarded in Session 1 Arena) and its two neighboring Adjacent Zones. Note that we consider together the two Adjacent Zones—bordering the Alternate Zone—in this analysis (rather than the usual one) because no reward had yet been delivered in Session 2 Arena. Therefore, from the participants' perspectives, there would be no difference between the two Adjacent Zones at this point. In this analysis, four participants who collected the first coin within 5 seconds of the trial were excluded.

Statistical modeling

Data analysis was conducted in R (version 4.1.2). Learning measures, and memory integration and differentiation were analyzed using linear mixed effects modeling with the lme4 package (version 1.1.27)[6]. The fixed factors of interest included Delay Condition (30min, 3h, and 27h) and Session (1 vs. 2), except when memory reactivation and perseveration were tested (only Session 2 data were analyzed). We also included arena shape (circle vs. square) as a covariate and a random intercept of Participant. This model was specified as preregistered, except for the inclusion of participants' Session 1 learning as a covariate. However, we also performed the analyses exactly as preregistered (i.e., without this covariate), and our conclusions about condition differences in memory differentiation and integration were the same; see **Supporting Results**). The *p* values were obtained using the lmerTest package and Satterthwaite's degrees of freedom method (version 3.1.3)[7], and pairwise comparisons were performed using the emmeans package (version 1.7.0)[8]. In cases where there was only one observation from each participant, linear models were conducted, and Session 1 learning was included as a covariate.

Bayesian analyses (brms package version 2.16.3)[9] were performed to estimate the strength of evidence for the null hypothesis when statistically non-significant condition differences were found with frequentist models. These Bayesian models included the same model structures. We used a weakly informed prior distribution as recommended by [10], to concentrate on possible values while also not being overly restrictive. For variables with a known range, we used a

normal distribution where all possible data fell within one standard deviation of the distribution. For example, the measurement for memory integration or differentiation always falls within (-1, 1), and therefore, condition difference in memory integration or differentiation would fall within (-2,2). A normal distribution with a mean of 0 and standard deviation of 2 (i.e., normal (0,2)) was selected as its weakly informed priors in this case. When a variable has an extremely wide range, we normalized all the variables before the calculation of the Bayes Factor, and then used the generic weakly informative distribution of normal (0, 1) as a prior distribution of effect sizes. For example, theoretically, difference in BIC between sigmoid and linear models can range from negative infinity to positive infinity; we therefore used the normal (0, 1) in this case. However, when we instead used the default flat distribution embedded in the brms package, our crucial conclusions regarding the temporal delay effects on memory differentiation and integration did not change, although the estimated strength of evidence for null effect did depend on the selection of prior distribution (see **Supporting Results** for details).

Supplementary Measures

After the main experiment, participants completed a separate virtual navigation task meant to assess individual differences in navigation, followed by a series of questionnaires:

- <u>Virtual navigation task.</u> The task was loosely modeled after the procedure of the Morris Water Maze[11–13]. Instead of searching for a platform in a circular pool, participants searched for a trophy in a pentagonal arena situated within a virtual room. The spatial cues in this task included a shelf, a bookcase, a window, and a door. Unlike the main experiment, the spatial cues were not positioned at cardinal directions. Participants were first presented with a visible trophy and instructed to navigate to the trophy's location to collect it. They then completed six trials in which they were told to navigate to an invisible trophy that was in the same location. As in the main experiment, participants started in a different location on each trial. A trial terminated once participants successfully located the trophy. In the final trial, unbeknownst to participants the trophy had been removed, and participants were simply given one minute to search for it. For the trials when the trophy was present, we measured the amount of time taken to find the trophy as an index of participants' spatial navigation ability. For the last trial, we calculated the proportion of time spent in the reward zone. Here, a reward zone was defined as a circular area where the center was the location of the trophy and the radius was the same as in the main experiment.

- Questionnaires. To examine the influence of individual differences in spatial navigation and circadian rhythms on learning and memory integration, at the end of the test session participants completed the Santa Barbara Sense of Direction Questionnaire[14], Navigational Strategies Questionnaire[15], the Morningness-Eveningness Questionnaires[16], along with a survey that included questions on their video game experience and strategies while performing the learning and test tasks.

Group differences in these measures (**Table S1**, **Fig S2**) and their associations with participants' learning and integration and differentiation of memories (**Table S3**) were examined using linear models. Briefly, while some of these metrics showed significant associations with task performance, none reliably differed as a function of delay condition.

References

1. Zeithamova D, Preston AR. Temporal Proximity Promotes Integration of Overlapping Events. J Cogn Neurosci. 2017;29: 1311–1323. doi:10.1162/jocn_a_01116

2. Yetton BD, Cai DJ, Spoormaker VI, Silva AJ, Mednick SC. Human Memories Can Be Linked by Temporal Proximity. Frontiers in Human Neuroscience. 2019;13. Available: https://www.frontiersin.org/articles/10.3389/fnhum.2019.00315

3. Lakens D. Performing high-powered studies efficiently with sequential analyses. European Journal of Social Psychology. 2014;44: 701–710. doi:10.1002/ejsp.2023

4. Alsbury-Nealy K, Wang H, Howarth C, Gordienko A, Schlichting ML, Duncan KD. OpenMaze: An open-source toolbox for creating virtual navigation experiments. Behav Res. 2022;54: 1374–1387. doi:10.3758/s13428-021-01664-9

5. Steemers B, Vicente-Grabovetsky A, Barry C, Smulders P, Schröder TN, Burgess N, et al. Hippocampal Attractor Dynamics Predict Memory-Based Decision Making. Current Biology. 2016;26: 1750–1757. doi:10.1016/j.cub.2016.04.063

6. Bates D, Mächler M, Bolker B, Walker S. Fitting Linear Mixed-Effects Models using lme4. arXiv; 2014. doi:10.48550/arXiv.1406.5823

7. Kuznetsova A, Brockhoff PB, Christensen RHB. lmerTest Package: Tests in Linear Mixed Effects Models. Journal of Statistical Software. 2017;82: 1–26. doi:10.18637/jss.v082.i13

8. Lenth R. emmeans: Estimated Marginal Means, aka Least-Squares Means. R package version 1.4.3.01. 2019 [cited 20 Aug 2021]. Available: https://CRAN.R-project.org/package=emmeans

9. Bürkner P-C. brms: An R package for Bayesian multilevel models using Stan. Journal of statistical software. 2017;80: 1–28.

10. Lemoine NP. Moving beyond noninformative priors: why and how to choose weakly informative priors in Bayesian analyses. Oikos. 2019;128: 912–928. doi:10.1111/oik.05985

11. Moffat SD, Resnick SM. Effects of age on virtual environment place navigation and allocentric cognitive mapping. Behavioral Neuroscience. 2002;116: 851–859. doi:10.1037/0735-7044.116.5.851

12. Moffat SD, Kennedy KM, Rodrigue KM, Raz N. Extrahippocampal Contributions to Age Differences in Human Spatial Navigation. Cerebral Cortex. 2007;17: 1274–1282. doi:10.1093/cercor/bhl036

13. Morris RGM, Garrud P, Rawlins JNP, O'Keefe J. Place navigation impaired in rats with hippocampal lesions. Nature. 1982;297: 681–683. doi:10.1038/297681a0

14. Hegarty M, Richardson AE, Montello DR, Lovelace K, Subbiah I. Development of a self-report measure of environmental spatial ability. Intelligence. 2002;30: 425–447. doi:10.1016/S0160-2896(02)00116-2

15. Brunec IK, Robin J, Patai EZ, Ozubko JD, Javadi A-H, Barense MD, et al. Cognitive mapping style relates to posterior–anterior hippocampal volume ratio. Hippocampus. 2019;29: 748–754. doi:10.1002/hipo.23072

16. Horne JA, Östberg O. A self-assessment questionnaire to determine morningness-eveningness in human circadian rhythms. International Journal of Chronobiology. 1976;4: 97–110.
