# Supplementary material for: Time separating spatial memories does not influence their integration in humans
Source: PLoS One. 2023 Aug 10;18(8):e0289649. doi: 10.1371/journal.pone.0289649 (PMC10414573; doi:10.1371/journal.pone.0289649)
Supplement: S2 File — (PDF) [file pone.0289649.s002.pdf]

Supporting Tables and Figures

Table S1: No significant differences in demographics or individual difference measures by delay conditions

Condition	N (F, M)[1]	Age[2]	Navigation Strategy	Sense of Direction	Morningness-Eveningness[3]
30min	44 (22, 22)	22.79 (3.89)	-0.19 (1.02)	-0.11 (0.95)	0.07 (0.98)
3h	45 (33, 12)	24.25 (4.16)	0.02 (0.95)	0 (1.03)	0.08 (0.96)
27h	42 (26, 16)	24.36 (4.60)	0.18 (1.02)	0.12 (1.03)	-0.15 (1.07)

Notes: Navigation strategy, sense of direction, and Morningness-Eveningness (MEQ) were coded with standard rubric and normalized data are reported. No difference among the three groups of participants were found (navigation strategy: $F(2,128) = 1.51$, $p = .226$; sense of direction: $F(2,128) = 0.55$, $p = .579$; MEQ: $F(2,128) = 0.71$, $p = .494$). [1]Number of participants in each delay condition who self-reported their sex as female (F) and male (M). No participants in our sample reported being of another sex or declined to respond to this question. [2]Two participants (one in 30min condition, one in 3h condition) provided unreasonable values for their year of birth and were not included in the analysis of age. [3]Local time of experimental sessions was not recorded and therefore we were not able to determine whether or not a session occurred during a participant's peak time of the day.

Table S2: Differences in time spent in different types of zones during Direct test under each delay condition

Delay	Session	Zones Compared	*beta*	*SE*	*Z*	*p*	*p.sig*
30min	1	Target vs. Alt	0.18	0.03	6.48	< .001	***
30min	1	Alt vs. Adj	0.12	0.03	4.19	< .001	***
30min	1	Adj vs. Ctrl	0.02	0.03	0.78	.438	ns
3h	1	Target vs. Alt	0.21	0.03	7.97	< .001	***
3h	1	Alt vs. Adj	0.18	0.03	6.60	< .001	***
3h	1	Adj vs. Ctrl	0.02	0.03	0.76	.447	ns
27h	1	Target vs. Alt	0.07	0.03	2.52	.012	*
27h	1	Alt vs. Adj	0.23	0.03	8.13	< .001	***
27h	1	Adj vs. Ctrl	0.01	0.03	0.20	.841	ns
30min	2	Target vs. Alt	0.35	0.03	12.86	< .001	***
30min	2	Alt vs. Adj	0.11	0.03	4.12	< .001	***
30min	2	Adj vs. Ctrl	0.01	0.03	0.35	.724	ns

3h	2	Target vs. Alt	0.33	0.03	12.22	< .001	***
3h	2	Alt vs. Adj	0.11	0.03	3.91	< .001	***
3h	2	Adj vs. Ctrl	0.06	0.03	2.26	.024	*
27h	2	Target vs. Alt	0.45	0.03	15.80	< .001	***
27h	2	Alt vs. Adj	0.08	0.03	2.81	.005	**
27h	2	Adj vs. Ctrl	0.02	0.03	0.86	.389	ns

Notes: Alt = Alternate; Adj = Adjacent Zone; Ctrl = Control Zone. ****p* < .001; **p* < .05; ns: not significant. See **Fig 2A** for visualization.

Table S3. Spearman correlations between individual difference measures and learning, memory differentiation and memory integration

Individual Difference Measure		Learning	Memory Differentiation	Memory Integration
Independent Virtual Navigation Task	Trophy Present and Visible	-0.04	0.09	-0.01
	Trophy Present and Invisible	**-0.18***	**-0.16~**	**0.15~**
	Trophy Absent	**0.20***	**0.25****	**-0.17***
Navigation Style		**0.18***	0.11	-0.12
Sense of Direction		0.14	0.09	-0.03
Morningness-Eveningness		0.03	-0.04	0.09

Notes: Three measures were taken from the independent virtual navigation task, including the time taken to find the visible trophy, time taken to find the invisible trophy, and the proportion of time spent in the Target zone when trophy was absent. Note that for the first two metrics, better performance corresponds with lower values (quicker searches), whereas for the final metric better performance corresponds with higher values (more time spent in the Target zone). Navigation style, sense of direction, and morning-eveningness are measured through questionnaires. Values in cells are Spearman correlation coefficients, with reliable associations denoted in bold and with ***p* < .01; **p* < .05; ~*p* < .10

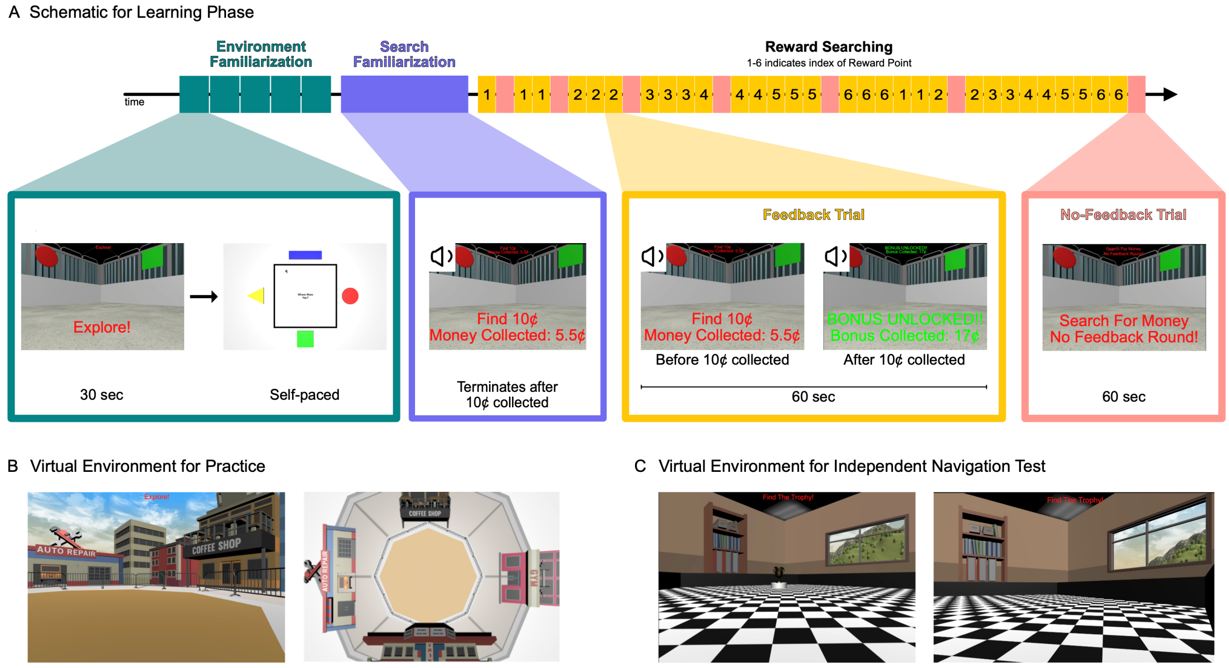

Fig S1. (**A**) Schematic depiction of the learning phase, showing the Environment Familiarization (teal), Search Familiarization (purple), and Reward Searching phases (yellow for feedback trials; peach for no-feedback trials). In the Environment Familiarization phase, the small triangle (top left quadrant) indicates the participant's current location in the arena. In the Reward Searching phase, no-feedback trials became decreasingly frequent over time. Top row shows timeline, with individual boxes representing separate trials. Numbers on yellow blocks indicate the index of the reward points that was reinforced during that particular block. Bottom row with pop-outs shows screenshots from each phase. Text feedback was small and appeared at the top of the screen in the real experiment. For legibility, we also include the text in larger font at the bottom of each screen in this figure. (**B**) Virtual environments used for practice test from a first-person (left) and aerial (right) view. (**C**) Virtual environments used for the independent navigation test from the first-person perspective, with (left) and without (right) the trophy visible. Environments shown in B and C were meant to be distinct from those used in the main memory experiment.

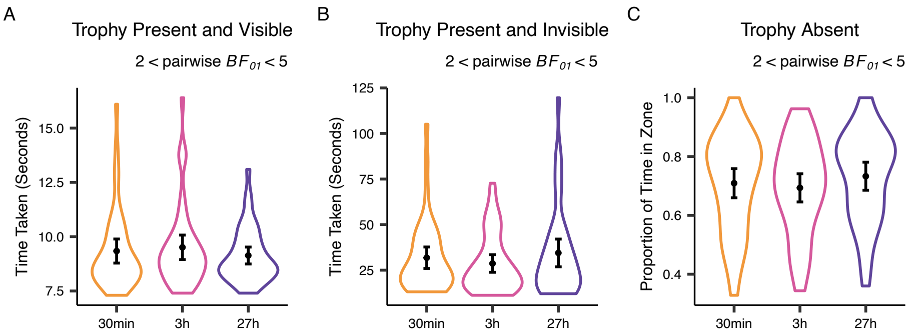

Fig S2. Performance on independent virtual navigation task. No group differences were found in any of the three types of trials: (**A**) Trophy Present and Visible ($F_{(2,128)}$ = 0.54, p = .583; main effect BF_{01} = 15.63, 2 < pairwise BF_{01} < 5), (**B**) Trophy Present and Invisible ($F_{(2,128)}$ = 0.90, p = .407; main effect BF_{01} = 10.08, 2 < pairwise BF_{01} < 5), and (**C**) Trophy Absent ($F_{(2,128)}$ = 0.67, p = .511; main effect BF_{01} = 13.79, 2 < pairwise BF_{01} < 5). This pattern of results suggests that there were no overall differences in virtual navigation ability across our delay groups.

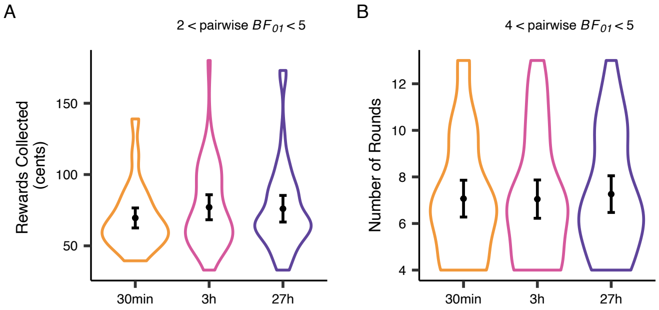

Fig S3. Performance on the practice task that occurred prior to the main memory experiment. There were no differences in performance across delay conditions in either (**A**) the total amount of rewards collected ($F_{(2,126)}$ = 0.97, p = .382; main effect BF_{01} = 10.06, 2 < pairwise BF_{01} < 5), or (**B**) the number of rounds completed to achieve the performance criterion ($F_{(2,126)}$ = 0.09, p = .912; main effect BF_{01} = 23.43, 4 < pairwise BF_{01} < 5). Again, this suggests that there were no differences in navigation performance generally across delay groups.

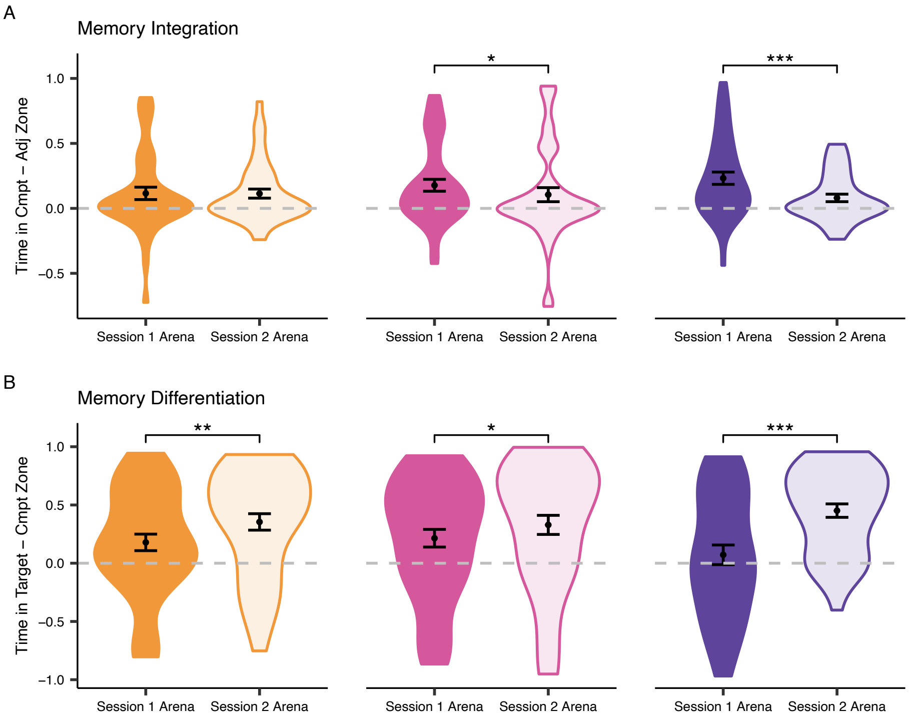

Fig S4. Memory integration (**A**) and differentiation (**B**) during the Direct test for arenas learned during Session 1 (dark) and Session 2 (light), respectively. *p < .05, **p < .01, ***p < .001

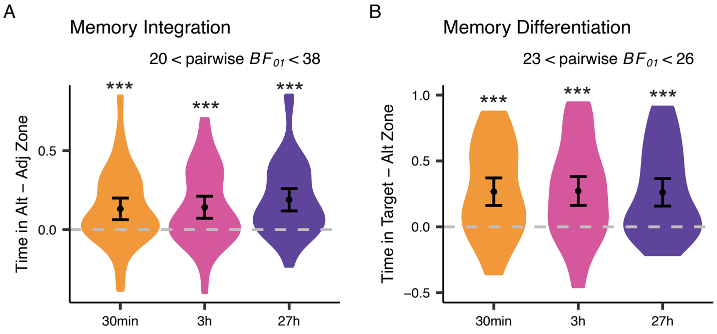

Fig S5. Memory integration (**A**) and differentiation (**B**) during the Direct test including participants who swapped the reward locations of the two arenas. The effect of temporal delay on memory integration (χ^2 (2) = 1.42, p = .493; main effect BF_{01} = 965.17; 20 < pairwise BF_{01} < 38) or differentiation (χ^2 (2) = 0.16, p = .925; main effect BF_{01} = 716.72; 23 < pairwise BF_{01} < 26) remained the same as in our primary analysis.

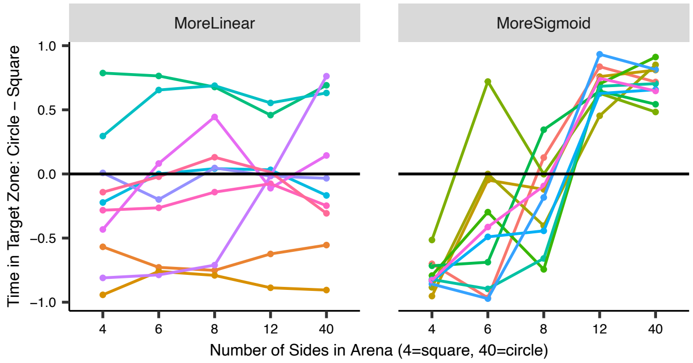

Fig S6. Search patterns of participants who showed the most linear and the most sigmoid shapes in the Transfer test. Each line represents one participant (n=10 per graph).

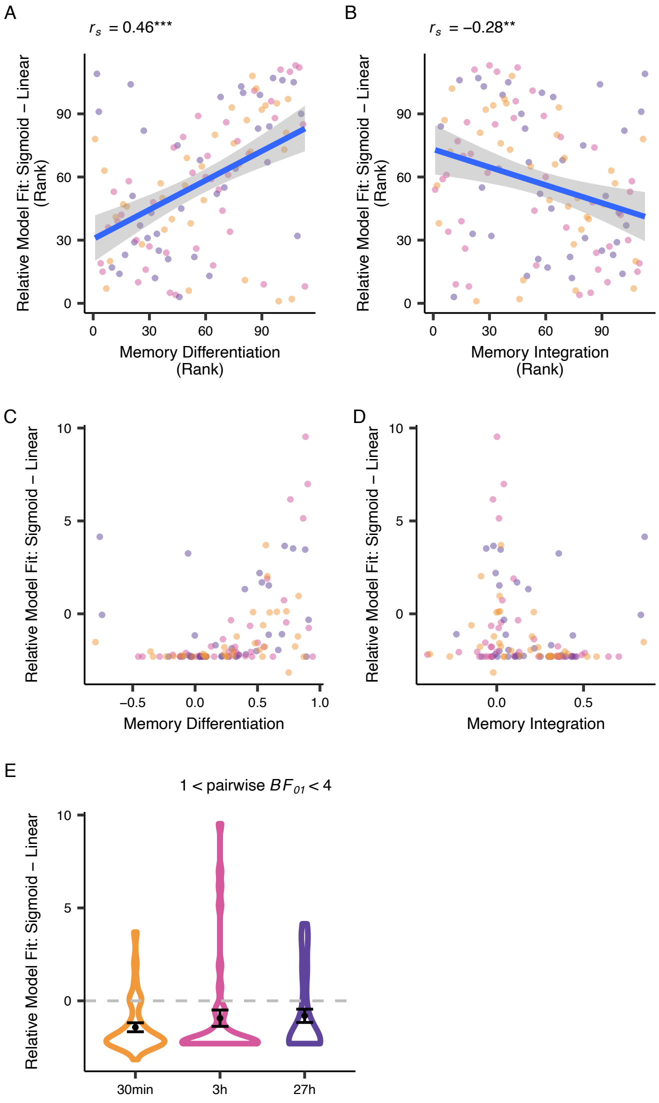

Fig S7. Transfer test including both influential points and participants who made swap errors. (**A-B**) Spearman correlation between relative model fit and memory differentiation and integration. (**C-D**) The same measures in the raw scales. (**E**) Temporal delay did not modulate relative model fit ($F_{(2, 109)}$ = 0.73, p = .483; main effect BF_{01} = 10.87; 1 < pairwise BF_{01} < 4). **p<0.01, ***p < .001.

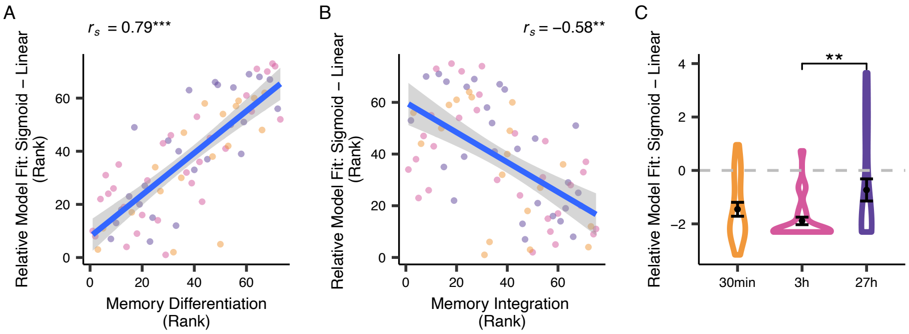

Fig S8. Transfer test restricting to participants who remembered both arenas. (**A-B**) Model comparison of Transfer test behaviour (y-axis) are positively correlated with memory differentiation (Target – Alternate Zone time) and negatively correlated with memory integration (Alternate – Adjacent Zone time) in the Direct test (x-axes). Note that ranks are shown rather than values to mirror the Spearman correlation approach. (**C**) Relative fit of sigmoid vs. linear models (y-axis) varied as a function of temporal delay (x-axis), driven by the 27h group's showing more sigmoid pattern than the 3h group. Positive values reflect superior fit of sigmoid over linear models; negative values the inverse. Model fit differences did not differ across delay condition. $^{**}p<0.01$, $^{***}p < .001$.

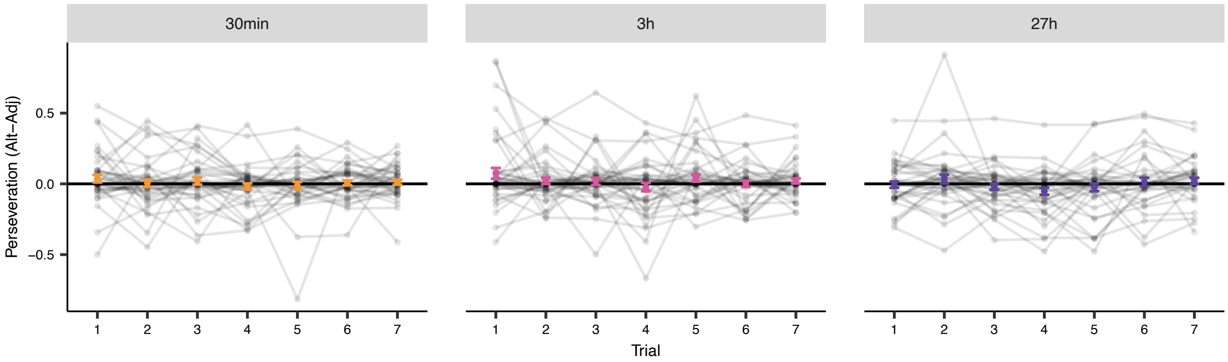

Fig S9. Perseverating on Session 1 Locations during Session 2 Learning. Perseveration score (y-axis; TIZ_{Alt}-TIZ_{Adj}) as a function of trial number (x-axis) during Session 2. Values above zero indicate that the participant was continuing to search in the location that had previously been reinforced in Session 1. Black semi-transparent lines represent individual participants; coloured dots and error bars are means and 95% confidence intervals across the group for 30min (orange, left); 3h (pink, middle); and 27h (purple, right) delay conditions.
