# Supplementary material for: Time separating spatial memories does not influence their integration in humans
Source: PLoS One. 2023 Aug 10;18(8):e0289649. doi: 10.1371/journal.pone.0289649 (PMC10414573; doi:10.1371/journal.pone.0289649)
Supplement: S3 File — (PDF) [file pone.0289649.s003.pdf]

Supporting Results

Preregistered statistical models without Session 1 Learning as a covariate in the analysis of Direct test (memory differentiation and memory integration)

Our preregistered did not include Session 1 learning as covariate in the analysis of memory integration and memory differentiation. Performing the analyses as preregistered, however, did not change the results. Participants showed more evidence of memory integration when in the arena they learned first (β = 0.07, SE = 0.02, t = 3.68, p < .001), and Delay did not influence memory integration (χ^2 (2) = 0.91, p = .635; main effect BF_{01} = 1547.77; 29 < pairwise BF_{01} < 43). For memory differentiation, Session 2 memories were more differentiated than Session 1 memories (β = 0.22, SE = 0.03, t = 6.78, p < .001), and again Delay did not influence memory differentiation (χ^2 (2) = 0.02, p = .991; main effect BF_{01} = 821.38; 25 < pairwise BF_{01} < 28).

Strength of evidence for null effect of delay using the default flat distribution as prior distribution

Although a flat distribution is not recommended in Bayesian modeling[1], we report estimation of evidence strength for null effect of temporal delay.

Memory differentiation (Direct test):

Main effect BF_{01} = 30.08; 4 < pairwise BF_{01} < 6

Memory integration (Direct test)

Main effect BF_{01} = 52.59; 5 < pairwise BF_{01} < 9

Relative model fit (Sigmoid vs. linear; Transfer test)

Main effect BF_{01} = 1.62; 0.7 < pairwise BF_{01} < 2

Memory reactivation (Session 2 learning)

Main effect BF_{01} = 49.29; 5 < pairwise BF_{01} < 7

Memory perseveration (Session 2 learning)

Main effect BF_{01} = 15.00; 1 < pairwise BF_{01} < 7

References

1. Lemoine NP. Moving beyond noninformative priors: why and how to choose weakly informative priors in Bayesian analyses. Oikos. 2019;128: 912–928. doi:10.1111/oik.05985
