# Supplementary material for: Time separating spatial memories does not influence their integration in humans
Source: PLoS One. 2023 Aug 10;18(8):e0289649. doi: 10.1371/journal.pone.0289649 (PMC10414573; doi:10.1371/journal.pone.0289649)
Supplement: Attachment — Submitted filename: Responses.docx [file pone.0289649.s004.docx]

**RESPONSE TO REVIEWER COMMENTS**

*We thank the Reviewers for their valuable and thoughtful comments on our original submission. By addressing their comments, we believe our revised manuscript has improved technical and interpretive clarity, along with a more thorough exploration of the data. The following manuscript additions are among the most substantial we have made: several new analyses that address potential confounds or previously unexplored patterns in our data, further details about the experimental procedure and stimulus norming, and a new discussion of limitations. Together these changes speak to the relationship between integration and interference, the role of memory precision in our findings, and the relation among learning speed, perseveration, and our observation of a recency bias. We thank the Reviewers for bringing our attention to the missing information as well as several important limitations that warrant discussion.*

*The Reviewers also had a number of other comments and questions, all of which we address in this revision as detailed below. Reviewer comments appear in normal font, and our responses are in italics. Thank you again for the helpful feedback!*

**Reviewer #1**

Note: For the question above, "Have the authors made all data underlying the findings in their manuscript fully available?" I said "no" because the OSF project they link to requires me to request access before I can view it. But the dataset does appear to have been deposited, though I can't actually see it and will not request access in order to avoid breaking reviewer anonymity.

*So sorry for this oversight! We have now ensured that our OSF projects, including the full datasets, are publicly available.*

Summary

The authors introduce a paradigm designed to bridge between human and rodent work examining how memories acquired in different environments are stored, and how integration and differentiation of environments in memory affects behavior. In contrast to rodent work that has generally focused on fear conditioning, the authors use an appetitive learning paradigm where participants learn a set of locations that are rewarded upon navigating to them. Participants learned about reward locations in two arenas with the same global cues but a different shape of enclosure. The authors focus on two main measures of behavior in unrewarded trials: (1) discrimination, the time spent visiting the rewarded locations specific to the current arena compared to locations that were rewarded in the other arena, and (2) integration, the time spent visiting rewarded locations specific to the other arena, relative to a location that is equidistant from the rewarded locations of the current arena. The authors also examine reward-seeking behavior in novel arenas of different shapes, to measure generalization of behavior to new environments. They find that participants with more evidence of discrimination in the main environments also show a predicted pattern of results consistent with behavior being guided by one environment or the other, but not both. In contrast to their main predictions, the authors find that the time delay between environments (30 minutes, 3 h, or 27 h) does not affect memory integration, as assessed by their environment confusion measures. They conclude that there are boundary conditions on the effect of delay on memory integration, which may relate to paradigm or species differences compared to previous work.


Evaluation

The authors address an important topic of temporal delay effects on memory representation that have been raised by recent research. The manuscript makes good use of study design and pre-registered statistical analyses. The main finding of a null effect of temporal delay on memory integration appears to be well established. Many questions remain about what the specific boundary conditions are on temporal delay effects, but the manuscript provides a valuable contribution in exploring the effect under different conditions.

*Thank you for this careful summary and overall positive evaluation of our paper. We are glad to hear the Reviewer appreciated the study design, pre-registered analytic plan, and strength of statistical evidence. We agree very much with the Reviewer that there remains much to be understood about the boundary conditions on temporal delay effects, and we hope that our work will inspire their further exploration!*

There is a focus throughout on very general mechanisms of integration and differentiation that I believe should be qualified to reflect the assumptions underlying those theoretical conclusions. In particular, effects that are here called "integration" have previously been labeled as reflecting "interference", and may reflect retrieval rather than purely reflecting memory storage, as "integration" is often understood. The terminology used should be clarified, and more discussion is necessary to make clear what behavioral effects have been shown here compared to what theoretical conclusions are being inferred from the results. As discussed below, some results are difficult to interpret, and may have alternate interpretations that should be considered.

*We thank the Reviewer for encouraging us to be more concrete in our use of terminology, and to clarify the mapping between our observed behavioural effects and the theoretical conclusions. We also now (in this response letter, as well as in the revised manuscript) consider the alternate interpretations referenced by the Reviewer—either through additional analyses, direct acknowledgment of limitations in the manuscript, or a combination of both. For more details, including our thoughts on the Reviewer’s point about integration versus interference and storage versus retrieval (detailed in Major issues comment #1), please see our responses to the Reviewer’s more specific comments below. Thank you again for raising these important points.*

Major issues

1. What is meant by "integration" is somewhat unclear. Memory integration, wherein two memories are represented similarly to one another, has previously been proposed as an important factor in determining behavior. As noted in the introduction, there is evidence that memory integration, at the level of individual engrams, is a factor in determining fear expression in rodents. Here, however, there is no measurement of memory engrams; instead, the manuscript reports evidence of interference effects, which are interpreted as evidence for integration. Depending on your working definition of "integration", this may be reasonable, but integration seems likely to be taken by some readers to mean something very specific about memories for the two environments being coded by overlapping engrams. However, that need not be the case here; global cues could trigger retrieval of either environment, causing interference, without there needing to be any overlap between engrams. It's reasonable to discuss potential implications of the results for memory integration (particularly given the main predictions of the effect of delay on engram integration), but it's also important to be clear when discussing the results that interference effects may also arise from other mechanisms without there needing to be engram overlap.

*We thank the Reviewer for raising this excellent point and encouraging us to sharpen the interpretive and theoretical clarity of our writing. The Reviewer is correct that the goal of our design was to focus on a very particular form of memory integration: Specifically, one in which two experiences are stored in a fused memory representation at the cognitive or neural levels. Our reasons for this focus are twofold. First, and as the Reviewer noted, our central research question about memory delay was inspired by rodent research demonstrating that this variable impacts the integration of neural engrams. Second, we ultimately intend to compare our human work with rodent models of memory—and of course in rodents, engrams and their behavioural significance can be measured directly. However, at the same time, we fully agree with the Reviewer that other mechanisms, such as retrieval-based processes in which interference arises between competing memories, could also contribute to the behaviours observed here. On the Reviewer’s recommendation, we have taken several steps to address this point.*

*First, when introducing memory integration in the relevant rodent work on p. 3, we clarify our definition of integration as follows:*

*For example, one study [4] showed that extinguishing the response to one fear-conditioned stimulus can transfer to another when the initial learning was separated by a 6- but not a 24-hour (h) delay. Such generalization (or false memory of fear for a never-conditioned stimulus, depending on your perspective) is emblematic of a type of memory integration in which experiences become fused into one memory representation. Hereafter, we refer to this fusing as “integration.”*

*Second, we now explicitly connect behavioural generalization with integrated memory representations when introducing our task objectives as: “be[ing] sensitive to the generalization and blurring thought to result from fused memory representations” on p. 5.*

*Third and lastly, we have added a new paragraph to the Discussion (p. 24-25) that unpacks the different forms of integration and interference that could contribute to behaviour in our task. Specifically, we now directly acknowledge the alternate possibility noted above by the Reviewer that retrieval-based interference might also contribute to our findings:*

*All this reasoning is centered on the type of integrative encoding mechanisms identified in the related rodent studies [3,4]—in which related experiences are encoded in overlapping sets of neurons—but the performance of our human participants may have been influenced by multiple integrative and interference processes. Indeed, developing clear operational definitions of integration—and generalization more broadly—has been a theoretical thorn in the side of our field [65] because multiple mechanisms dictate how memories interact at both the time of encoding and retrieval. Broadly construed, behaviours reflecting memory integration could be supported by three encoding processes [37]: (1) fusing experiences within one representation, potentially at the cost of idiosyncratic experiential details [66,67], (2) explicitly linking two separately represented experiences [68,69], for example, by building a complex situation model [70], and (3) separately encoding memories that can later be recombined at the time of retrieval [71,72]. With the goal of targeting the first mechanism (i.e., the one most relevant to the neurobiological impact of delay), we used a task and behavioural indices that did not distinguish between the generalization and interference that can result from a fused representation. However, other types of integration and/or interference could contribute to task performance. For example, participants could receive high scores on our integration index by storing separate memories of each session that then compete with each other at the time of retrieval. Indeed, this type of retrieval competition has been shown to underlie many demonstrations of memory interference [73–75]. Notably, a recent fMRI study showed that this kind of retrieval competition depends on how memories are reactivated and integrated during related learning [76]. Thus, while our focus has been on integrative encoding mechanisms, the role of interconnected interference during retrieval should not be discounted.*

*We hope the Reviewer agrees that these changes collectively serve to both clarify the scope of integration mechanisms targeted by our approach, while also cautioning the reader to consider the many other processes that could contribute to performance. Thank you again for this helpful suggestion!*

2. The main measure of differentiation is ambiguous, as it may reflect at least two different sources of individual variability: (1) interference between environments, potentially due to memory integration (as hypothesized in the manuscript), and (2) spatial precision of memories within a given environment. If spatial precision is higher for a given participant, such that they consistently respond close to the rewarded area and not further from the rewarded area, then the TIZtarget-TIZalternate difference will tend to be higher compared to participants with lower spatial precision. In that case, some of the variability in individual differences would reflect variability in spatial precision rather than differentiation between the two environments. The integration measure does not suffer from this issue, as both zones of interest there are equidistant from the target zone.

The strong correlation with the fit of a sigmoidal model reduces this concern, as it's reasonable to think there is a theoretical link between those measures, but still, memory precision (in terms of specific spatial location) could also be correlated with attractor-like dynamics across environment morphs, in addition to memory differentiation (in terms of whether the environments are confused). The (likely small) potential effect of spatial precision on the differentiation measure should be discussed. Optionally, there might be some way to control for it by measuring the spatial precision of responses and controlling for variance in spatial precision when measuring individual differences in differentiation.

*We thank the Reviewer for raising this limitation of our differentiation metric. We agree it would be helpful to understand how precision contributes to our findings. Indeed, in our original submission, we were concerned that individual differences in spatial memory abilities (e.g., Session 1 learning, performance on an independent water maze control task) could obscure our ability to observe the impact of delay in our between-subject design. Of these indices, we only observed group differences in Session 1 learning, which (because Session 1 occurs prior to the manipulation) must reflect pre-existing group differences. Accordingly, in the original submission (and retained in the revision), all of the analyses we report in the main text include Session 1 learning performance as a covariate in the statistical models, to control for these differences. Therefore, the null effects we observe in differentiation (and integration) as a function of our time manipulation are after accounting for variability in Session 1 performance—or what could be conceptualized as memory precision. We hope that the Reviewer agrees that these controls address the role of precision to the extent possible in our paradigm. However, of course, these precision proxies are imperfect. As such, we also took the Reviewer’s first suggestion and added a full paragraph to the Discussion to describe this limitation (p. 25-26):*

*Relatedly, delay’s null impact on memory integration and differentiation may reflect limitations in our ability to infer these memory representations from behaviour. In our Direct test, we defined differentiation as spending more time in the Target than Alternate zone, and integration as spending more time in the Alternate than Adjacent zone. Therefore, memory precision could contribute to our differentiation scores, as* differentiation *requires participants to both remember the reward locations of different arenas and to do so with enough precision to not stray into the Alternate zone. By contrast, integration would not be influenced by precision. There are several reasons to believe that this imbalance did not overly influence our primary results, though. First, the delay groups did not differ in their expression of memory precision in our independent virtual navigation task, suggesting similar spatial memory precision across conditions. Therefore, it is unlikely that individual differences in memory precision would have impeded our ability to detect true delay-dependent differences in integration or differentiation. Second, we also statistically controlled for individual differences in spatial memory ability by using Session 1 learning (which did show modest delay differences) as a covariate in all analyses reported in the main text. Third, we purposefully, used large zone definitions, encompassing nearly a full quadrant of the arena. Accordingly, even imprecise memories would be sufficient to get participants to the target zone. Lastly, this differentiation index was strongly correlated with sigmoidal fits in the Transfer test (which would be less directly influenced by precision), suggesting that they track the same underlying construct.*

3. The higher accuracy on no-feedback trials in the second environment is interpreted as potentially being evidence for integration, i.e., that previous experience with the first environment leads to improved learning. However, that finding also seems compatible with participants improving at the task through other means, such as learning how to orient using wall landmarks, or getting better at seeking reward efficiently. To show clearer evidence of a transfer effect, ideally you would compare performance on a second environment that shares global cues with the first to performance in a new environment that does not share those cues, and show that there is improved performance only when global cues are shared with the first environment. The alternative interpretation of environment performance differences, i.e., that performance may be affected by general task learning rather than necessarily reflecting environment transfer, should be discussed.

*We thank the Reviewer for highlighting this important miscommunication; Reviewer 2 also made a similar point in their 5^th^ comment. We completely agree with both Reviewers’ reasoning. In fact, we did not intend to imply that superior Session 2 performance was clear evidence of transfer, but instead only meant to note that it has been interpreted this way in related research in rodents. We agree that our original phrasing “a transfer effect attributed to memory integration in related rodent work [4,43]” was ambiguous. To clarify our intended meaning, we have replaced it on p. 13-14 with “Similar observations of transfer effects have been attributed to memory integration in related rodent work [3,18], but we note that it could also reflect learning general skills required by the task, like using cues to navigate or developing strategies to find hidden rewards.” We also clarified the ambiguity in interpreting performance improvements when reporting how they do not interact with the delay manipulation on p. 14. Thank you again for bringing this issue to our attention, and we apologize for the lack of clarity in our original submission.*

4. It's unclear that a linear model fit to performance across the different environment morphs would necessarily indicate integration. The curves reported in the supplement that best fit a linear model tend to be flat, indicating that the balance of circle and square reward region searching did not change between the different morphs. That pattern is potentially consistent with integration, especially for participants with no bias toward one reward region or the other, but many participants seem to be biased to look for reward near one environment's target region or the other. This seems consistent with them simply remembering one environment and responding in accordance with that, rather than necessarily retrieving an integrated representation of both environments. The inclusion criteria for this analysis only required above-chance memory for at least one of the two arenas (page 18), which allows for the possibility that included participants only remembered one of the arenas.

*We thank the Reviewer for raising this point. In response to the Reviewer’s concern, we have conducted an additional analysis with more stringent inclusion criteria: Specifically, we asked whether our main findings held when including only those participants with above-chance memory for both arenas. Seventy-eight participants met this criterion. Under these stricter inclusion conditions, we again found that the linear model provided the better fit to performance (Wilcoxon signed-rank test, V = 2433, p < .001). We also found that the correlations with differentiation (r _s_ (71) = 0.79, p < .001) and integration (r_s_ (73) = -0.58, p < .001) as assessed on the Direct test held, as before. However, in contrast to our main analysis, which required participants to remember just one arena to be included, here we found a significant effect of temporal delay (F(2,70) = 4.74, p = 0.012). This overall effect was driven by a significantly better linear fit in the 3h (n = 28) than the 27h group (n = 26; β = 1.22, SE=0.40, t = 3.06, p = .003; other pairwise comparisons were not significant), consistent with our initial predictions. We are hesitant to strongly interpret this finding in the face of strong evidence for the null in all our other preregistered and exploratory analyses. In particular, we see two competing possible explanations. On one hand, temporal delay manipulations only influence the integration of particularly strong memories. Alternatively, the delay effect may reflect a survival bias; this stricter criterion may have been particularly restrictive for the 27h group (whose Session 1 memory was tested at the longest delay), leaving only participants with particularly differentiated memories. Thus, this group could appear to have the most differentiated memories due to the subsampling rather than the delay itself. We have revised the Supplementary Information (Supplemental* ***Fig S8****), Results (p. 17-18), and Discussion (e.g., p.22 and p. 23) to incorporate these new findings and potential interpretations. Thank you again for this comment!*

Minor issues

The deposited data on OSF currently cannot be accessed without requesting access from the authors.

*Our apologies for this oversight!* *We have changed the setting to allow for public access of the data (URL:* [*https://osf.io/se8t7/*](https://osf.io/se8t7/)*)*

Page 13: The preregistration for wave 2 (<https://osf.io/gnxpt>) is currently not publicly accessible.

*We apologize for this. We have ended the embargo status of the Wave 2 preregistration to allow for public access.*

Page 14: More detail should be given about how the study was run, especially given the online nature of the study. How was the study run on participants' computers? For example, was the study run using a web browser? If so, were there any requirements for which browser participants must use, or requirements for the computer? Are there any details of how you monitored performance remotely that might be useful for other researchers conducting online studies?

*We thank the Reviewer for pointing out these missing details in our procedure description. We have added the suggested information to the “Data acquisition and preprocessing” section in the Supplementary Information on p. 2:*

*Participants completed the tasks by running lab-developed applications on their own computers, as described in [40]. This required us to be more cautious compared to running in-lab studies. Prior to the first session, we (1) asked participants to make sure their computers had available storage of at least 300MB to download and run the applications; and (2) checked in with participants about their computer specifications to make sure our applications were likely to work on their devices. Based on our experiences, applications typically ran smoothly on computers with at least 4GB of memory. While most devices that met these requirements worked well, we did encounter situations where a computer met the memory requirement, but the processor was too old to run the applications, and therefore, a session had to be terminated. During the sessions, participants shared their screens via Zoom so that experimenters could monitor the whole process and provide help when needed. They (1) instructed participants to close applications that were irrelevant to the experiment to maximize memory allocation for the experimental applications and to minimize distractions at the beginning of a session; (2) guided participants to download and install the specific version of our experiment application that was compatible with their operating systems (Windows or Mac OS); (3) checked in with participants during the practice task and breaks to make sure they (a) did not experience lags, which were typically related to poor computer performance, and (b) were remaining engaged in the tasks.*

Page 15: Please give some more detail about the norming data. How many participants were in the norming study? How did they rate perceptual discriminability? Were they shown first-person images of the environments, or maps, or did they navigate the environments?

*Thank you for this suggestion. We have now incorporated more details on the norming data as requested to p. 9:*

*Norming study for morph arena development and selection. To select morph shapes, we conducted an online norming study using Inquisit 5 Software on Amazon’s Mechanical Turk. Participants (n=40) were sequentially presented with a pair of environment screenshots from a 1^st^ person perspective. Half of the pairs were identical; the other half only differed in enclosure shape (4-12 sides). Participants were asked to indicate if the images were identical or different after seeing both presented for 1s each, separated by a 1s coloured mask. We then identified the optimal spacing of morphs such that their perceptual discriminability was roughly equated. This procedure led to the selection of morphs with 6, 8, and 12 sides.*

Page 15: More detail should be specified for the environment familiarization phase. How was location displayed on the aerial view? From Figure S1, it looks like a triangle was shown, but the screen cap is small and hard to see; in any case, this would be good to mention in the methods text also. Was starting location randomized? If so, were there any constraints on the randomized placement?

*Thank you for these suggestions. To answer the Reviewer’s specific questions, the triangle on the aerial view schematic in Figure S1 indeed represents the participant’s location in the arena. Participants started from the center of the arena and moved the triangle using the arrow keys until they reached the location that they had last occupied in the 3D environment. When navigating the 3D environment from the 1^st^ person perspective, starting location within the arena and initial head direction were both random (no constraints). We have added relevant details to the Environment Familiarization section on p. 10 and refer readers to Figure S1 to facilitate understanding. We have also revised the Figure S1 legend caption to explain the tiny triangle. Thank you again for pointing out these missing details!*

Figure 4: "Cmpt" (short for competing?) should be "Alt".

*We thank the Reviewer for pointing out this error. We have fixed this in Figure 4.*

What was the delay between waves 1 and 2, and when were they collected? This might be relevant especially if data were collected during the early stages of the pandemic, when conditions were changing rapidly and a delay between data collection of different conditions could be a concern. Of course, the various control analyses you included to test for cohort differences would reduce any such concern.

*Thank you for raising this concern. The delay between the two waves of data collection was about two months, and both waves occurred mid-pandemic.* *More specifically,* *Wave 1 occurred between July 21 and December 23, 2020, and Wave 2 between February 22 and June 5, 2021. We have added the dates for both waves of data collection to the “Sample Size Determination” section on p. 8. We agree with the Reviewer that data collected during the earliest days of the pandemic might have been particularly difficult to interpret, as would a very long delay between the two data collection waves; however, we believe our timing and relatively short delay would likely not meet either of these criteria. As noted by the Reviewer, we are also encouraged by the lack of group differences on the various questionnaires and the independent spatial navigation task. As such, we hope that including the data collection dates in the paper will sufficiently address the Reviewer’s concern. Thank you again!*

Optional suggestions

Memory integration could lead to intrusion of the non-current reward locations, as predicted in the manuscript, but I wonder if there could also be a more subtle bias introduced by memory integration. If memories for the environments are truly merged, could this lead to participants searching for reward in a "compromise" area between the two locations in the different environments? Looking for this could involve testing to see if responses in one environment are closer to the alternate zone compared to the adjacent zone, to see if there is a subtle nudge in the direction of the alternate reward locations, even when the responses are not in the alternate zone itself.

*We thank the Reviewer for this suggestion; it is a very interesting idea! However, looking for such subtle differences in the locus of participants’ search behaviour would be challenging in our design because the particular reward locations shifted across learning. Specifically, rewards were presented at six different (but partially overlapping) “points” during learning, which together made up the overall reward distribution centered on the middle of the target quadrant. Given our task structure, then, the most recently rewarded location was never in the exact center of the Target zone, but rather was always physically closer to one of the two neighbouring zones (depending on whether the participant was in the circle or square arena). For this reason, we believe our design is not optimal for testing the Reviewer’s ideas about small biases in search behaviour, in which participants’ search centers shift slightly toward the Alternate zone. However, we agree with the Reviewer that these are very interesting questions and should be addressed in future work on this topic.*

*More broadly, thank you again for your time and insightful comments!*

**Reviewer #2**

This manuscript details a well-designed and interesting study examining how temporal proximity influences the integration or differentiation of memories. The task is designed to translate paradigms from rodent research to humans, a commendable and important endeavor, and the results add to the growing literature understanding the boundaries on encoding processes that lead to integration/differentiation. I do have several questions and comments, as detailed below:

*Thank you for the positive evaluation of our work! We have responded to each of your specific points below.*

1. Clarification on methods/terminology. How overlapping were the 6 ‘points’ pulled from the gaussian distribution? Were participants instructed that the rewards would not always be in the same location on each exploration opportunity? Were the quadrants of rewards in the circle and square arena always in the same relation to one another (e.g., in Figure 1A, they appear to be in adjacent quadrants, raising the possibility that it may be easier to learn the second arena if the reward distribution center was closer to the first)

*We appreciate the questions and the Reviewer’s keen eye for identifying design details that warrant clarification. Turning first to questions about reward locations, to minimize differences in participant experiences, we used the same reward locations across all participants (e.g., square arenas were rewarded in the top right quadrant for all participants). The locations presented in Figure 1A reflect the exact locations of the six points in each arena and are rendered to scale. All points had at least some overlap with others in the set of six, but the degree of overlap varied. We revised the legend for Figure 1A on p. 7 to clarify this, and also added a note in the text to this effect (p. 9-10). Since all participants experienced the same reward locations, yes, the two rewarded zones were always in adjacent quadrants. This was by design—we wanted to encourage the integration across sessions (while allowing for differentiation), so we placed the reward zones in close (but non-overlapping) locations. We clarified this design decision further on p. 8.*

*As for the question about instructions: Participants were not explicitly told if the rewards would always appear in the same location. Our reason for this choice was to mimic mice experiments in which, of course, such explicit instruction is not possible. However, participants could have anticipated the shifting reward locations. Specifically, passing the practice session (as a criterion for inclusion) required them to successfully search for rewards across shifting points. Therefore, participants could have inferred that the main experiment would have the same structure. We have changed the Reward Searching section on p. 10 to reflect this. Thank you again for the questions!*

2. Were there any order effects in learning between the two arenas (e.g., was it easier to learn the circle before the square, or vice versa?)

*Thank you for this question.* *To assess this possibility, we asked whether our main learning metric (Time in Target Zone on the final two no-feedback trials) varied as a function of the order of learning (i.e.,* whether the circle or square arena was experienced first)*. Our analysis showed that the order of learning did not influence the learning performance (t(129) = 0.32, p = .749). We now report this on p. 13.*

3. Was the extent to which participants showed a recency bias for session 2’s location on the direct test related to how quickly participants acquired the session 2 location or generally how quickly participants learned the point locations during reward search? I’m wondering if there are individual differences that could predict and unpack this bias more.

*This is an interesting idea—perhaps the observed recency bias wasn’t just driven by the recency of the Session 2 learning, but instead, the superior learning that occurred during Session 2? To test this idea, we first needed a measure of learning speed. Each session included seven no-feedback trials that could be used to estimate learning; performance on feedback trials is a poor indicator of learning because participants could use real-time feedback to maximize performance. In the original submission, we used the final two no-feedback trials to operationalize final learning. Here, we explored two possibilities for operationalizing learning speed: (1) the number of no-feedback trials taken to reach the predefined threshold (30% of time spent in Target Zone [TIZ_target_]), or (2) the average TIZ_target_ across all no-feedback trials. Unfortunately, there was little difference across individuals when we considered option 1: 81.7% of participants reached the threshold in the very first no-feedback trial (range: 1-4). However, participants did have more variability in their average TIZ_target_ (no participants had the same value; mean=0.71, SD=0.18, range=0.345-0.995), so we chose option 2. Note that all participants experienced the same sequence of trials and the same trial duration, making the average TIZ_target_ a reasonable approximation for how quickly and effectively participants learned reward locations. With this metric, we saw learning speed improve from Session 1 to Session 2 (F(1, 128) = 40.55, p < 0.001; note this is conceptually similar to the data we originally reported in the manuscript and displayed in Figure 1C, though that data considered only the final two no-feedback trials, whereas here we consider all trials). Mirroring the final learning effects reported in the manuscript, when learning was considered in this way, learning speed was not influenced by temporal delay in general (F(2, 128) = 1.57, p = 0.212), or during Session 2 specifically (F(2,128) = 1.09, p = 0.34). Moreover, there was also no interaction between temporal delay and session (F(2, 128) = 0.07, p = 0.936), further supporting the conclusion that the transfer in learning across sessions did not depend on delay. Note that we did not add these statistics to the revised manuscript as they lead to the same interpretation as the originally reported final learning analyses.*

*More germane to the Reviewer’s question, we then asked whether individual differences in learning speed related to the observed recency bias. We found that while the recency effect (difference in TIZ_target_ observed on the direct test between the Session 2 and Session 1 arena) was not correlated with overall learning speed (r_s_ (126) = 0.05, p = .580), but it did show a trend for an association with Session 2 learning speed (r_s_ (126) = 0.16, p = .079). Moreover, the recency bias was correlated with the difference in learning speed across sessions (Session 2 – Session 1; r_s_ (126) = 0.26, p = .003). These results are generally consistent with the idea that the recency bias, at least in part, reflects the superior learning of Session 2 reward zones, and not just that Session 2 was learned more recently. We now report the difference score result, which we think is most interpretable, on p. 15. Thank you again for the suggestion!*

4. Somewhat related to question 3, the authors could also examine the speed of learning for the second arena compared to the first, rather than just the integration behavior on the direct test. Does the extent to which participants continue to perseverate on the learned reward location in the first arena have any relationship with later integration (or differentiation) on the direct test? In addition to the authors hypothesis that reactivation could facilitate ‘bridging’ across memories and thus support integration, it could also be the case that the longer participants continue to search in the prior location without finding a reward could serve to highlight differences across the arenas. It would be interesting to see how learning ability may mediate the relationship between reactivation and integration or differentiation. The authors do note generally that the 3hr group was faster on both arenas, but what about the other groups?

*We appreciate the Reviewer’s insight here and agree that there may be complex relationships between reactivating Session 1 memories and forming integrated vs. differentiated representations. In the original manuscript, we only addressed the idea by measuring Session 1 reactivation during the new environment exploration (prior to learning Session 2 reward locations) and correlating this reactivation score with later integration and differentiation. To address the Reviewer’s question about perseveration, we took a related approach but now using no-feedback trials during Session 2 learning. Specifically, we measured perseveration as being the difference between time spent in the Alternate (previously rewarded) zone minus the time spent in the Adjacent zone (TIZ_Alt_ - TIZ_Adj_). A more positive value would mean greater perseveration. Note that while perseveration would likely yield poor performance, it uses different zones than the learning speed measure (TIZ_Target_) described above, and therefore the two measures need not be tightly related.*

*The figure above (also* ***Figure S9****) shows the time course of perseveration in each delay condition. Combining groups and trials, we did not see a reliable perseveration effect (β = 0.01, 95% CI = [0, 0.02]). However, perseveration did decay over trials (F(6, 768) = 2.70, p = 0.013), and was significantly above zero on the first no-feedback trial* (*β = 0.04, 95% CI = [0.01, 0.06]), suggesting that it was present but relatively short-lived. Accordingly, we used the perseveration score on the first no-feedback trial as our index of perseveration.*

*Next, we addressed the Reviewer’s question as to whether perseveration is related to differentiation or integration across individuals. Interestingly, we found that this index of perseveration was positively correlated with subsequent integration (r_s_ (126) = 0.21, p = .018) and negatively correlated with differentiation (r_s_ (126) = -0.18, p = .046) on the Direct test. However, it was not correlated with evidence for attractor states in the Transfer test (r_s_ (109) = 0.16, p = .102). These relationships did not significantly differ by delay condition (Fs < 1.2, ps > .30). This suggests that continued reactivation and use of Session 1 reward locations early during Session 2 bridges across memories in support of integration, regardless of the time separating learning experiences. We now report these findings in detail in the revised Results (p. 20-21) and Discussion (p. 24), and in the newly added* ***Figures 5*** *and* ***S9****. Thank you for the great suggestion!*

*As for the final question, we believe the Reviewer is asking whether the transfer effect (better final learning for Session 2 than Session 1) is present for each delay condition separately, as well as whether the 30min and 27h delay conditions were different from one another during either Session 1 or Session 2. We found that when considered separately, there was a significant improvement from Session 1 to Session 2 for the 27h group (27h: β = 0.11, SE = 0.03, t = 3.15, p = .002) that was not reliable in the other two groups (30min: β = 0.05, SE = 0.03, t = 1.51, p = .134; 3h: β = 0.05, SE = 0.03, t = 1.40, p = .166). However, the delay x session interaction was not significant, meaning that the improvement did not reliably differ by delay group. These statistics are now reported in the revised manuscript (p. 14). In addition, 30min and 27h groups were not different from one another during either Session 1 (β = 0.01, SE = 0.04, t = 0.14, p = .886) or Session 2 (β = -0.05, SE = 0.04, t = -1.25, p = .214; see p. 14).*

5. I found the claim in the first paragraph of the results section (on the initial learning performance), that the findings of stronger learning by the end of session 2 constitute a transfer effect attributed to memory integration in rodent work to be somewhat unclear – are the authors suggesting this pattern has been shown in studies that show shared neural populations? Otherwise, why wouldn’t this simply be a function of task practice? Perhaps consider operationalizing the term or explaining the findings on the transfer effect more thoroughly in the introduction.

*We thank the Reviewer for highlighting this important miscommunication; Reviewer 1 also made a similar point in their 3^rd^ comment. We completely agree that our original phrasing was ambiguous and problematic. Our intention was to simply note that superior Session 2 learning has been interpreted in this way in related research in rodents, but not to suggest it as our interpretation. To address this mistake, we replaced the original text, “a transfer effect attributed to memory integration in related rodent work [4,43]” on p. 13-14 with “Similar observations of transfer effects have been attributed to memory integration in related rodent work [3,18], but we note that it could also reflect learning general skills required by the task, like using cues to navigate or developing strategies to find hidden rewards.” We also clarified the ambiguity in interpreting performance improvements when reporting how they do not interact with the delay manipulation on p. 14.*

*We appreciate the Reviewer’s recommendation to expand on this related research in the Introduction, and if we did have a strong interpretation of the session differences, we would certainly follow it. However, since the new phrasing better achieves our goal of providing a little context while reporting a finding, we thought that expanding the Introduction around this logic was not necessary. After reading our clarification, if the Reviewer still sees the value in this, we would be happy to do so.*

6. Why include the two arenas again during the transfer test? Couldn’t this re-exposure serve as anchors that may bias behavior on the morphs, especially considering that they were also just tested on the direct test?

*Thank you for this question. Our main reason for including the two directly learned arenas in the transfer test is that we needed five points to compare sigmoid versus linear models. Moreover, it seemed inappropriate to use behaviour from a separate (direct) test in this analysis, and, as such, we reasoned that it was best to include the arenas from the learning tasks in the transfer test, intermixed with the three novel morphs. We have added this logic to the description of the Transfer test on p. 11 to clarify. Thanks again for pointing out this missing detail in our original description of the approach.*

*We are very grateful for your helpful comments—thank you!*
