# Supplementary material for: Time separating spatial memories does not influence their integration in humans
Source: PLoS One. 2023 Aug 10;18(8):e0289649. doi: 10.1371/journal.pone.0289649 (PMC10414573; doi:10.1371/journal.pone.0289649)
Supplement: Attachment — Submitted filename: Reviews_R2_ToSubmit.docx [file pone.0289649.s005.docx]

**RESPONSE TO REVIEWER COMMENTS**

*We appreciate the Reviewers taking the time to read our revised manuscript and are glad that they are happy with our revision. We also thank the Reviewers for bringing our attention to the following minor issues. Below please find our response to the comments. Reviewer comments appear in normal font, and our responses are in italics.*

Reviewer #1: My major concerns have been addressed by the manuscript revisions, through new analyses and discussion points. I note a few remaining minor concerns below.

*We are glad to hear that our new analyses and discussions have addressed your major concerns. Thank you for catching the minor issues to help us further improve the manuscript! Below please see our response to each individual point.*

Minor issues:

Page 6: The two preregistration links are the same. The link for wave 2 is shown twice, and the preregistration for wave 1 (https://osf.io/49dtx) is not shown here.

*Thank you for pointing this out! We have corrected the link for the wave 1 preregistration. Please see page 6 for the change.*

Figure 3 - In my copy of the PDF, there are no points shown for the scatterplots in panels B and C, only a regression line. These points were previously visible in the original version and appear to not have been modified in the revision, so this was not an issue for evaluating the revision.

*Thank you for noticing the figure presentation issue with Figures 3, 5, and S7.*

*We also noticed a similar issue with Figures S8 and S9. This unexpected issue seems likely related to the image type that we used and/or the file conversion. In this revision, we have changed the figures to EPS image files before inserting them into the Word document, and based on our tests, they work well with the submission system. However, please do let us know if the issue remains.*

Figure 5 - I also see no scatterplot points in my copy of the PDF for panels B, C, and D.

*Thank you for catching this! We believe it is resolved now. Please see our previous response regarding Figure 3 for details.*

Figure S4 - I believe "Cmpt" should be "Alt".

*Thanks again for the careful eye. We have fixed this by changing the title of y-axis of Figure S4. Please see page 13 of* ***Supplementary Information*** *for the change.*

Figure S7 - In my copy, I don't see any points in panels A, B, C, and D. These points were shown in the original manuscript, and the results seem to not have changed in the revision, so this was no an issue for evaluating the revision.

*Thank you very much for pointing this out. We believe it is resolved now. Please see our response regarding Figure 3 for details.*
