# Supplementary material for: Time separating spatial memories does not influence their integration in humans
Source: PLoS One. 2023 Aug 10;18(8):e0289649. doi: 10.1371/journal.pone.0289649 (PMC10414573; doi:10.1371/journal.pone.0289649)
Supplement: Attachment — Submitted filename: ResponsesToReviews.docx [file pone.0289649.s006.docx]

**RESPONSE TO EDITOR COMMENTS**

*Thank you again for bringing to our attention the errors that previously existed in our analysis. Below we respond (italics) to each specific Editor comment (normal font).*

Journal Requirements:

Please review your reference list to ensure that it is complete and correct. If you have cited papers that have been retracted, please include the rationale for doing so in the manuscript text, or remove these references and replace them with relevant current references. Any changes to the reference list should be mentioned in the rebuttal letter that accompanies your revised manuscript. If you need to cite a retracted article, indicate the article’s retracted status in the References list and also include a citation and full reference for the retraction notice.

*We confirm that reference list is complete and correct. We also ensured that none of the cited articles has been retracted using reference management software Zotero, which uses data from Retraction Watch.*

Additional Editor Comments:

1. Figure 3B – In the last round of review, one reviewer noted that the data points in the scatter plots were not visible. In the revised R2 version, the data points are now present. However, the data do not match what was submitted in the original submission (R0). At first glance, it appeared that the color coding simply changed, but this is not the only change/difference (although it appears to be one change). Most noticeable difference with respect to the data is the pink data point in the original version that is at coordinates ~(75, 5) that is no longer present in the revised version. In the revised version, **please explain why this image is no longer the same as well as the impact on the reported results.**

*Thank you again for noticing this error. The Editor is correct that there were changes in both the colour mapping as well as the data itself. Colour coding and labels were incorrect in R0 because of an R error in which conditions were ordered alphabetically rather than by delay duration. In our re-analysis and re-visualization of all data, we have ensured that the condition orders are now manually defined according to delay duration and are referenced throughout the analysis script. This error would impact data visualization only, and as such does not have any bearing on the statistics or conclusions (which were of course derived from the statistics themselves).*

*The difference in data points relates to the issue we described in the cover letter, in which outdated versions of data were included in some analyses. Specifically, data used for this analysis had been preprocessed using an old pipeline that had minor differences from the methods described in the manuscript, for example, how data was temporally resampled. This error ultimately affected which participants were deemed to have met our inclusion criteria based on learning for the Transfer test Analysis: As a reminder, we required that participants remembered at least one of the two arenas (as noted in the revised manuscript on p. 13). Ultimately, the outdated pipeline excluded slightly different groups of participants, causing discrepancies between the two versions of the figure referenced by the Editor. Of course, this error also had a (small) impact on the statistics relating to the Transfer test performance (p. 16-18). However, encouragingly, the results of our checking revealed that correcting this error did not have any substantive impact on our conclusions. As noted in the cover letter, one place in which our correction made a noticable difference is that now, there is a statistical trend when comparing the relative model fits between the 3h and 27h delay conditions (Figure 3D). We now make note of this in the paper (p. 17); however, given it is a trend in a follow-up pairwise comparison on a non-significant ANOVA, we suggest that the evidence is not strong enough to influence the main message of the paper. We have corrected all statistics (p. 16-18) and visualizations (Fig. 3) associated with this analysis; however, no changes to the discussion section were necessary.*

*Detecting this error prompted us to check whether incorrect versions of data were included in other analyses. Specifically, we have thoroughly checked all results by rewriting our scripts to seamlessly progress from raw data to statistical analyses and figures, finding one minor error in the selection of practice data trials that did not change the results of analyses. We then re-processed all raw data from scratch, double checked all reported statistics, and replaced all graphs with freshly analyzed data. For good measure, we also replicated all results across multiple computers. Lastly, we publicly posted raw and preprocessed data, the streamlined analysis scripts, and knit html documents that transparently displays all statistical results and figures produced by the script. Again, results did not change meaningfully after these corrections, aside from some statistical trends that were unrelated to our hypotheses becoming significant and vice versa.*

2. Figure 5, S8 and S9 - these were all added as part of R1 (not included in R0). In light of the discrepancy noted above for Figure 3, **please confirm that these images and the corresponding statistics are accurate.**

*Thank you for highlighting these figures as ones that might be potentially affected by colour mapping errors. We have thoroughly checked these and all other figures. In doing so, we identified that four supplemental graphs had colour mapping issues in some submissions: FigureS2-ABC (all null effects) had incorrect mapping in all submissions, and the colours used to visualize delays had incorrect mapping in the correlation displayed in Figure S8B in R2. These colour mapping errors did not influence the interpretation of results. We can confirm that all graphs now accurately display delay conditions according to the indicated colour mapping.*

3. Figure S7; Panel E – When reviewing the updated figures, I noticed that the color coding changed from the original version to R1 (remained the same from R1 to R2). Specifically, data originally labeled in R0 as the 30m group were re-labeled as 27h in R1, data originally labeled as 3h were re-labeled as 30mn in R1, and data originally labeled as 27h were re-labeled as 3h in R1. I didn’t see this change explicitly mentioned in the previous response document.  **Given the lack of a significant effect, it appears this change had no impact on the results. Can you confirm? And please also confirm that the most recent version of the figure (in R1 and R2) is indeed the correct one.**

*Yes, the most recent version is the correct one--thank you for catching this! As noted above, this was related to the colour mapping issue in which conditions were automatically ordered alphabetically rather than by duration in R0. To address this issue, we manually defined colour mappings and labels according to delay duration to verify that the most recent version of the figures has the correct colour coding. All figures were also recreated with the correct preprocessing and to reflect the correct groups of participants (related to point 1).*

4. Figure S7 Panels A-D and Figure 3B – The datapoints are now visible and match what was in the original version with no change to the corresponding results. Accordingly, issues surrounding these images are considered addressed.

*Thank you for verifying that the datapoints are now visible on your end. We appreciate it! Also as noted above, these figures additionally needed to be corrected to address the issue of reading in the wrong data.*

*Thank you again for bringing these important issues to our attention.*
